# Targeting cathepsin K diminishes prostate cancer establishment and growth in murine bone

**DOI:** 10.1007/s00432-019-02950-y

**Published:** 2019-06-06

**Authors:** Weiping Liang, Fuhao Wang, Qiuyan Chen, Jinlu Dai, June Escara-Wilke, Evan T. Keller, Johann Zimmermann, Ni Hong, Yi Lu, Jian Zhang

**Affiliations:** 1 Key Laboratory of Longevity and Aging-Related Diseases, Guangxi Medical University, Ministry of Education, Nanning, 530021 Guangxi China; 2 School of Medicine, Southern University of Science and Technology, Shenzhen, 518055 Guangdong China; 3 Guangdong Provincial Key Laboratory of Cell Microenvironment and Disease Research, Shenzhen, 518055 Guangdong China; 4 State Key Laboratory of Oncology in South China, Collaborative Innovation Center for Cancer Medicine, Sun Yat-sen University Cancer Center, Guangzhou, Guangdong China; 5 Department of Pathology and Internal Medicine, University of Michigan, Ann Arbor, MI USA; 6 Novartis Pharma Ltd., Basel, Switzerland; 7 Department of Urology, University of Pittsburgh, Pittsburgh, PA 15240 USA; 8 Present Address: Polyphor Ltd, Hegenheimermattweg 125, 4123 Allschwil, Switzerland

**Keywords:** Cathepsin K, Prostate cancer, Skeletal metastasis, Animal model

## Abstract

**Background:**

The processes of prostate cancer (PCa) invasion and metastasis are facilitated by proteolytic cascade involving multiple proteases, such as matrix metalloproteinases, serine proteases and cysteine proteases including cathepsin K (CatK). CatK is predominantly secreted by osteoclasts and specifically degrades collagen I leading to bone destruction. PCa and breast cancer preferentially metastasize to the bone. Importantly, CatK expression level is greater in PCa bone metastatic sites compared to primary tumor and normal prostate tissues. However, the underlying mechanism of CatK during PCa metastases into the bone remains to be elucidated. We investigated the functional role of CatK during the PCa establishment and growth process in the murine bone.

**Methods:**

CatK mRNA expression was validated by RT-PCR, protein expression by immunoblotting in PCa LNCaP, C4-2B, and PC3 cells as well as in PCa tissues. Its protein production was measured using ELISA assay. The effect of both knockdowns via siRNA and CatK inhibitor was compared in regard to PCa cell invasion. We further studied the dose-dependent CatK inhibitor effect on conditioned media-induced bone resorption. In setting up an animal model, C4-2B cells were injected into the tibiae of SCID mice. The animals treated with either vehicle or CatK inhibitor for 8 weeks at the time of tumor cell injection (tumor establishment model; protocol I) or 4 weeks after tumor cell injection (tumor progression model; protocol II) were applied to histological and histomorphometric analyses.

**Results:**

We confirmed CatK expression in PCa LNCaP, C4-2B, and PC3 cells as well as in PCa tissues. Furthermore, we observed the inhibitory effects of a selective CatK inhibitor on PCa cell invasion. The CatK inhibitor dose-dependently inhibited PCa-conditioned media-induced bone resorption. Upon injection of C4-2B cells into the tibiae of SCID mice, the selective CatK inhibitor significantly prevented the tumor establishment in protocol I, and reduced the tumor growth in bone in protocol II. It also decreased serum PSA levels in both animal models. The inhibitory effects of the CatK inhibitor were enhanced in combination with zoledronic acid (ZA).

**Conclusion:**

The selective CatK inhibitor may prevent the establishment and progression of PCa in bone, thus making it a novel therapeutic approach for advanced PCa.

## Introduction

The skeleton is the most common site of metastatic disease after lung and liver. Bone metastasis is more prevalent than primary bone tumors and remains a major cause of cancer death (Jayarangaiah and Theetha Kariyanna 2018). Prostate, breast, lung, and nasopharyngeal carcinoma frequently metastasize to skeletons. Particularly in prostate cancer (PCa), bone metastases tend to be the only site of metastasis (Jimenez-Andrade et al. 2010). Studies have shown that ~ 90% of men with advanced PCa will develop bone metastases (Bubendorf et al. 2000; Gandaglia et al. 2015). PCa skeletal metastases are most often radiographically characterized as osteoblastic (increased bone mineral density) lesions (Logothetis and Lin 2005), despite high osteoclast activity as indicated by elevated serum and urinary markers of bone resorption (Clarke et al. 1991; Sano et al. 1994; Takeuchi et al. 1996). However, histological evidence shows that PCa skeletal metastases form a heterogeneous mixture of osteolytic and osteoblastic responses (Berruti et al. 1996; Roudier et al. 2003). Emerging evidence proved that osteoblastic metastases form on trabecular bone at sites of previous osteoclastic resorption and that such resorption may be required for subsequent osteoblastic bone formation (Carlin and Andriole 2000; Zhang et al. 2001). The mechanisms through which PCa cells promote bone resorption and subsequent woven-bone formation remain poorly understood.

Previous studies have shown that osteoclast activities are important for the development of bone metastases in several cancer types including breast, lung, and PCa (Fazzalari et al. 2001; Loberg et al. 2005). Increased osteoblast activity likely favors PCa cell growth in bone. Factors such as osteonectin, osteopontin, osteocalcin, and bone sialoprotein secreted by osteoblasts have been shown to affect different PCa cell functions (Ortiz and Lin 2012). Osteopontin, for example, affects PCa cell proliferation, invasion, and intravasation potential (Khodavirdi et al. 2006). Aiming to block tumor expansion in bone, anti-resorptive approaches have been developed in cancer animal models, such as administration of bisphosphonates, i.e., zoledronic acid (ZA) or parathyroid hormone-related protein (PTHrP) neutralizing antibody (Guise et al. 1996; Sasaki et al. 1995). In PCa skeletal metastasis animal models, we and others have demonstrated anti-resorptive agents such as soluble receptor activator of NF-kappaB (sRANK) (Zhang et al. 2003), osteoprotegerin (OPG) (Zhang et al. 2001), and overexpression of OPG successfully diminished the tumor growth in bone (Corey et al. 2005). In clinical application, ZA effectively reduces metastasis-related bone pain and skeletal complications in patients with metastatic PCa and breast cancer (Saad et al. 2004), whereas more than 30% of patients will experience at least one skeletal complication after ZA therapy within a 2-year period, and ZA-unrelated serious events (Khalafallah et al. 2018; Rosen et al. 2004). Therefore, new effective treatments are urgently needed for patients with bone metastases and those at high risk of developing bone metastases.

Cathepsin K (CatK) is a cysteine protease member of the cathepsin lysosomal protease family. It is identified as an osteolytic and protease enzyme, degrades bone matrix proteins including type I collagen, osteopontin, and osteonectin (Garnero et al. 1998; Novinec and Lenarcic 2013). It is highly expressed in osteoclasts, but other family members cathepsins B, L and S are expressed at negligible levels (Drake et al. 1996). Patients with pycnodysostosis, a disease characterized by abnormal bone remodeling (Markatos et al. 2018), carry mutations in the *catK* gene (Gelb et al. 1996) and mice with a null mutation in the *catK* gene develop osteopetrosis of the long bones and vertebrae (Saftig et al. 1998). CatK knockout mouse is capable of mitigating high-fat diet-induced cardiac hypertrophy and contractile dysfunction, indicating that cathepsin K contributes to the development of obesity-associated cardiac hypertrophy (Hua et al. 2013); CatK knockout also alleviates age-related decline in cardiac function via suppressing apoptosis (Hua et al. 2015). Since CatK possesses one of the highest matrix degradation activities with higher efficiency than other cathepsins and metalloproteinases (MMPs) (Chapman et al. 1997; Garnero et al. 1998), it has been implicated to play an essential to role in disease cases involving bone and cartilage destruction (Borel et al. 2012), even tumor invasion (Schmit et al. 2012; Sinha et al. 1995; Szpaderska and Frankfater 2001; Yan et al. 1998) and rheumatoid arthritis (Dodds et al. 1999; Hummel et al. 1998).

CatK was also reported in breast cancer cells capable of causing bone resorption (Littlewood-Evans et al. 1997). Its mRNA was detected in PCa cell lines and in primary PCa and metastases (Brubaker et al. 2003). Importantly, CatK expression in bone metastases was significantly greater than primary PCa, while CatK expression in normal prostate tissues was negative (Brubaker et al. 2003) suggesting that CatK may play an important role in PCa skeletal metastases. Many selective CatK inhibitors have been developed to potently inhibit osteoclast resorption both in vitro and in vivo (Le Gall et al. 2007; Lu et al. 2018). In this study, we report that CatK contributes to PCa-induced osteoclast activity at bone metastatic sites, and inhibition of CatK by a selective inhibitor may prevent the establishment and progression of PCa in bone.

## Materials and methods

### Cell lines and cell culture

Human prostate cancer cell lines PC3 and LNCaP cells were purchased from the American Type and Culture Collection (ATCC, Manassas, VA) and were cultured in RPMI 1640 medium. C4-2B cells (Dianon, Oklahoma City, OK) were derived from the parental LNCaP cells but with characteristics of skeletal metastasis. They were maintained in T medium (80% DMEM, 20% Ham’s F12 medium [Invitrogen, Carlsbad, CA], 5 µg/mL insulin, 13.6 pg/mL triiodothyronine, 5 µg/mL transferrin, 0.25 µg/mL biotin, and 25 µg/mL adenine [Sigma, St. Louis, MO]). Primary murine bone marrow cells (MBMC) were cultured in the αMEM medium. All cell cultures were supplemented with 1% penicillin/streptomycin (Invitrogen, Carlsbad, CA) and 10% fetal bovine serum (FBS) (HyClone, Pittsburgh, PA). Prostate epithelial cells (PrEC) are human epithelial cells (Cambrex, Walkersville, MD) and were cultured using PrEGM BulletKit media (Cambrex). All cells were maintained in a 37 °C incubator equilibrated with 5% CO_2_.

### Animals

Male SCID mice (Charles River, Wilmington, MA) at 6 weeks of age were housed under pathogen-free conditions in accordance with the NIH guidelines. The animal protocol was approved by the Institutional Animal Care and Use Committee, University of Pittsburgh.

### CatK inhibitor

CatK inhibitor used in this study was kindly provided by Novartis Pharma Ltd (Basel, Switzerland). The structure of CatK Inhibitor has previously been reported (Grabowska et al. 2005). The dosages were chosen as 50 mg/kg/day and 100 mg/kg/day in the in vivo study, based on the renal toxicity of dose 500 mg/kg/day in the rat, and preclinical efficacy and tolerability dose of 50 mg daily used in a human.

#### Conditioned media (CM)

CM was obtained from PCa cells as previously described (Lu et al. 2007a). Briefly, 2 × 10^6^ cells were plated in 10-cm tissue culture dishes for 12 h in RPMI 1640 with 10% FBS. The media were then changed to 10 ml of RPMI 1640 plus 1% FBS, and supernatants were collected 48 h later. To normalize for differences in cell density due to cell proliferation during the culture period, cells from each plate were collected and total DNA content/plate was determined. CM was then normalized for DNA content between samples by adding RPMI.

#### CatK mRNA expression and quantification

Total RNA was extracted from LNCaP, C4-2B, PC3 and PrEC cells using TRIzol reagent (Life Technologies, Gaithersburg, MD), then subjected to RT-PCR for detection Cat K mRNA. PCR primers used for detection of CatK consisted of sense 5′-CAG CAA AGG TGT GTA TTA TGA TGA AAG C-3′, and antisense 5′-ATG GGT GGA GAG AAG CAA AGT AGG AAG G-3′ resulting in a PCR product of 399 bp (Genebank accession no. X82153). Beta-actin cDNA was amplified as a control for RNA quality. The PCR products were subjected to electrophoresis on a 1.5% agarose gel, stained with ethidium bromide. For quantification of CatK mRNA expression, real-time RT-PCR was performed in an iCycler iQ multicolor real-time RT-PCR detection system (Bio-Rad, Hercules, CA) using the iScript one-step RT-PCR kit with SYBR Green (Bio-Rad). Melting curve analysis was performed to evaluate the purity of the PCR products. Triplicate samples were run for each primer set. The relative expression of CatK to GAPDH (as housekeeping gene control) was calculated using the ΔCT method as previously described (Lu et al. 2007b). CatK primers were: sense 5′-CAG CAG AGG TGT GTA CTA TG-3′ and antisense 5′-GCG TTG TTC TTA TTC CGA GC-3′. GAPDH primers were: sense 5′-CCA TGG AGA AGG CTG GGG-3′ and antisense 5′-CAA AGT TGT CAT GGA TGA CC-3′.

#### Immunoblot analysis

To evaluate the CatK protein expression in PCa cells, confluent LNCaP, C4-2B, PC3, and PrEC cells were washed with PBS and whole-cell lysates were prepared using standard procedure (Lu et al. 2007c). The protein concentration of the cell lysates was measured by BCA kit (Pierce, Rockford, IL) and proteins (40 μg/lane) were applied to SDS-PAGE followed by Western blot with rabbit anti-human CatK polyclonal Antibody (Santa Cruz Biotech, Santa Cruz, CA), rabbit anti-E-cadherin antibody (Cell Signaling Technology, Danvers, MA), rabbit anti-Vimentin antibody (CST), rabbit anti-Snail antibody (CST), rabbit anti-GAPDH antibody (CST) or mouse anti-human β-actin monoclonal antibody (Sigma). The antibody binding was revealed using an HRP-conjugated anti-rabbit IgG, or HRP-conjugated anti-mouse IgG1 (Santa Cruz) and enhanced chemiluminescence (ECL) blot detection system (Amersham Biosciences, Piscataway, New Jersey).

### ELISA

To evaluate CatK protein production by PCa cells, CM collected from LNCaP, C4-2B, PC3 and PrEC cell cultures were measured by CatK ELISA kit (Alpco, Windham, NH) following the manufacturer’s protocol. Total PSA levels in serum were determined using the Accucyte Human PSA assay (Cytimmune Sciences, College Park, MA). Means ± standard errors were calculated from triplicates.

### siRNA knockdown experiment

The designed CatK siRNA or scrambled siRNA (Santa Cruz) was transfected into C4-2B cells. Briefly, C4-2B cells were cultured in 6-well plates (3 × 10^5^/well) with antibiotic-free cell growth medium 1 day prior to transfection. For each transfection, 1 µg of siRNA duplex was diluted in siRNA transfection medium to a final volume of 100 µl. Six-microliter siRNA transfection reagent was diluted into 100 µl of siRNA transfection medium and added directly into the siRNA duplex solution. After 30 min incubation at room temperature, 0.8 ml of transfection medium was added to each tube containing the siRNA and transfection reagent mixture. The mixture was overlayed onto the cells which had been washed by siRNA transfection medium. Cells were incubated for 6 h at 37 °C and cell growth media containing 2 × serum and antibiotics were then added. The cells were incubated for an additional 24 h and the media were replaced with cell growth media for 48 h. Cell lysates were collected for Western blot to confirm the knockdown of CatK protein expression.

#### Cell proliferation

Cell proliferation was measured by a CellTiter 96 AQeous Non-Radioactive Cell Proliferation Assay (Promega, Madison, WI). Briefly, C4-2B cells were plated in 96-well plates at a density of 5000 cells/well in 200 µL of T medium with 5% FBS. After 12 h of culture, the media was changed to RPMI 1640 plus 0.5% FBS and a serial concentration (0–100 μM) of CatK inhibitor was added. Cells were incubated at 37 °C in a humidified 5% CO_2_ atmosphere for 24 h, then 20 µL of MTS/PMS solution was added. After incubation for 2 h at 37 °C, the absorbance of each well at 490 nm was recorded using an ELISA plate reader. Data represent the average absorbance of six wells in one experiment.

#### In vitro cell invasion assay

The in vitro invasion assay was performed using C4-2B cells in the presence or absence of CatK inhibitor. C4-2B cells transfected with CatK siRNA or control siRNA. The invasiveness of cells was evaluated in 24-well matrigel invasion chambers (BD Biosciences, Bedford, MA), as directed by the manufacturer (Lu et al. 2006). In upper compartments, a serial concentration of CatK inhibitor (0, 1, 10, 50, 100 µM) was added. The transwell chambers were incubated for 24 h at 37 °C in 95% air and 5% CO_2_. Cell penetration through the membrane was detected by staining the cells on the porous membrane with a Diff-Quik stain kit (Dade Behring, Newark, DE). Cells that had penetrated through the membrane were counted in five microscopic fields (at × 200 magnification) per filter. The invasiveness was defined as the proportion of cells that penetrated the matrix-coated membrane divided by the number of cells that migrated through the uncoated membrane (baseline migration). The results are reported as the mean of triplicate assays.

### In vitro bone resorption assay

CM (25%, v/v) from C4-2B cells was added to non-adherent primary murine bone marrow cells (1 × 10^5^ from C57BL/6 mouse femurs) that were seeded into the 24-wells of BD Biocoat Osteologic Bone cell culture system (BD Bioscience). It consists of sub-micro synthetic calcium phosphate thin films coated on to the culture vessels and on dentin slices in 96-well plate. Soluble RANKL (50 ng/ml) and M-CSF (10 ng/ml) were used as positive controls or different concentration of the CatK inhibitor was added. The osteoclast culture was maintained for 10 days and half of the media was changed every 3 days. Then the cells were fixed and stained for tartrate-resistant acid phosphatase (TRAP) (Kamiya Biomedical, Seattle, WA). Resorptive areas on the digital images of osteologic or dentin slices were measured using a BIOQUANT system (R&M Biometrics, Inc., Nashville, TN). Samples were evaluated in triplicates.

#### Experimental protocols for intraosseous tumorigenesis

The animal studies are summarized in Fig. 4a. In protocol I, mice were randomized to receive either the vehicle or CatK inhibitor (50 mg/kg/day as the low dose and 100 mg/kg/day as the high dose, orally, *n* = 10/group) at the time of C4-2B cell injection (3 × 10^5^ cells) and continued for 8 weeks. Before killing, the animals were anesthetized, and magnified flat radiographs were taken using a Faxitron (Faxitron X-Ray Corp, Wheeling, Illinois). In protocol II, 4 weeks after the injection of tumor cells, one group (*n* = 10/group) was killed as basal and other mice (*n* = 10/group) were randomized to receive vehicle, CatK inhibitor (50 mg/kg/day), zoledronic acid (ZA, 100 μg/kg) and CatK inhibitor combined with ZA, respectively. The tumors were allowed to develop for eight additional weeks. At killing, the blood samples were collected and measured for PSA levels. All of the major organs and lumbar vertebrae were harvested for histological analyses.

#### Histopathology and bone histomorphometry

Histopathology was performed as described previously (Zhang et al. 2003). Briefly, bone specimens were fixed in 10% formalin and decalcified in 10% EDTA for 6 days. After paraffin embedding, the specimens were sectioned (5 µM), followed by staining with hematoxylin and eosin (H&E). Non-stained sections were deparaffinized and rehydrated for staining of prostate-specific antigen (PSA) with anti-human PSA antibody using standard immunohistochemistry techniques. Staining for TRAP was performed on non-stained sections. Human PCa tissue microarray specimens with corresponding non-neoplastic tissues from ISU Abxis (Seoul, Korea) (Lu et al. 2006) were stained for CatK. For routine histopathology, soft tissues were preserved in 10% formalin, paraffin embedded, sectioned (5 μM) and stained with H&E. To evaluate xenografts proliferation, sections were deparaffinized, rehydrated and stained with Ki67 monoclonal antibody following a modified protocol (Salas et al. 2004). The Ki-67 labeling index (KI) was calculated as the percentage of positive tumor nuclei divided by the total number of tumor cells examined. At least 1000 tumor cells per specimen were examined in five randomly selected fields by light microscopy (× 400) by an investigator who was blinded to the animal groups as previously described (Wallner et al. 2006). Histomorphometric analysis was performed on a BIOQUANT system (BIOQUANT-R&M Biometrics Inc) as previously described (Zhang et al. 2003). Four discontinuous random regions of interest were examined within each tibia, without any information about the treatment group with magnification at × 100. The tumor volume was determined as the proportion of tumor area in the total nonmineralized portion of the bone.

### Bone mineral density analysis

Total bone mineral density and trabecular bone mineral density of tibiae were measured using peripheral quantitative computed tomography (pQCT) (Stratec, Ontario, CA). Three slices of the proximal metaphases region were scanned to obtain trabecular bone mineral densities.

### Subcutaneous tumors

As a parallel study for protocol I, C4-2B cells were resuspended in T media simultaneously with the intratibial injection. Two million cells were mixed 1:1 with Matrigel (Collaborative Biomedical Products, Bedford, Massachusetts), and injected into the right flank at 100 μl/site using a 23-g needle (*n* = 10/group). Subcutaneous tumor growth was monitored by palpation, and two perpendicular axes were measured. The tumor volume was calculated using the formula as: volume = length × width^2^/2.

### Statistical analysis

Statistical analysis was performed using Statview software (Abacus Concepts, Berkley, CA). Student’s *t* test was used to compare two groups, whereas ANOVA was used for initial analyses of comparing multiple groups. Fisher’s Least Significant Difference (LSD) test complemented the post hoc analyses. Differences with a *p* value < 0.05 were considered statistically significant.

## Results

### CatK is highly expressed in metastatic PCa cells

**Fig. 1:**
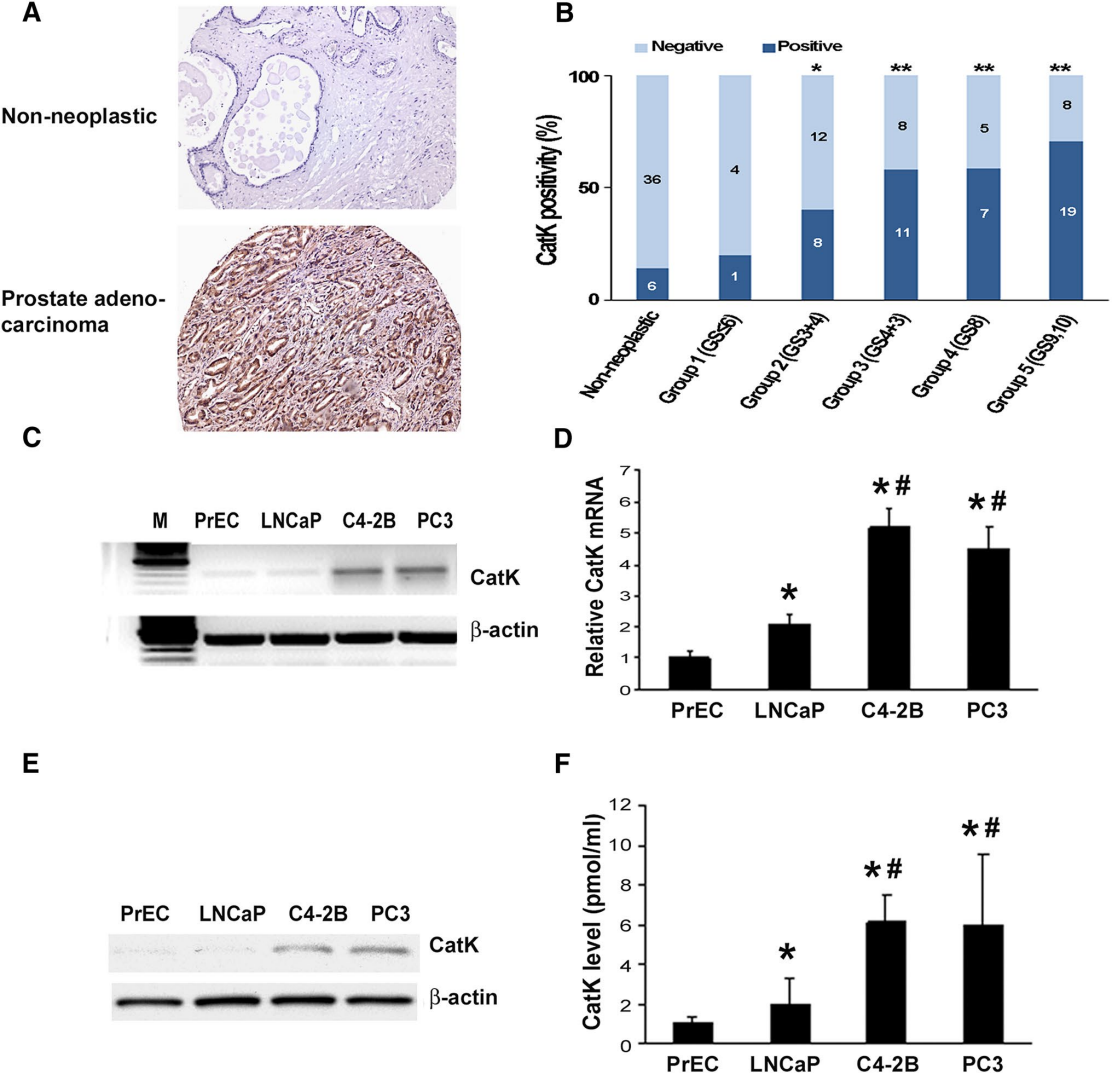
CatK mRNA and protein expression in PCa tissues and cell lines. **a** CatK expression in human PCa tissues. Immunohistochemical staining was performed for detection of CatK in human PCa and corresponding non-neoplastic tissues. **b** Quantitative evaluation of CatK expression in PCa. Dark blue bars represent case number of CatK-positive staining, and light blue bars show case number of CatK-negative staining. Statistically significant differences were noted between PCa and corresponding non-neoplastic tissues. **P < 0.001 compared to non-neoplastic tissues. **c** Total RNA was extracted from prostate epithelial cells (PrEC), LNCaP, C4-2B, and PC3 cells, then subjected to RT-PCR for the detection of CatK mRNA. PCR product of 399 bp is detected. **d** Quantification of CatK mRNA determined by real-time PCR. Internal control is β-actin. *P < 0.05 compared to PrEC; ^#^*P* < 0.01 compared to LNCaP cells. **e** To evaluate for CatK expression in the prostate cancer cells, confluent PrEC, LNCaP, C4-2B, and PC3 cells were washed with PBS and then lysed in RIPA buffer. Proteins were applied to SDS-PAGE followed by Western blot system with rabbit anti-human CatK polyclonal Ab, or mouse anti-human β-actin monoclonal Ab. The Ab binding was revealed using an HRP-conjugated anti-rabbit IgG, or HRP-conjugated anti-mouse IgG1 and enhanced chemiluminescence blot detection system. **f** To test CatK production in the PCa cell supernatants, CM collected from PrEC, LNCaP, C4-2B, and PC3 cell cultures were measured by an ELISA kit. *P < 0.01 compared to PrEC; ^#^*P* < 0.01 compared to LNCaP cells

To evaluate for expression of CatK in PCa, we performed immunohistochemistry on a tissue microarray (TMA). CatK immunohistochemical staining was at low levels in the normal prostate, increased in PCa tissues (Fig. 1a). We observed that 46 out of 83 (55.4%) PCa tissue specimens expressed CatK at heterogeneous levels. Specifically, CatK expression levels were elevated significantly along with WHO prostate cancer grading system (Fig. 1b). In contrast, 6 out of 42 (14.3%) non-neoplastic samples expressed CatK although at low levels. These results are similar as previously reported by Brubaker et al. (Brubaker et al. 2003). We also found that C4-2B cells (C4-2B cells were derived from LNCaP cells but with characteristics of bone metastasis) expressed higher levels of CatK mRNA than LNCaP and PrEC cells. C4-2B cells expressed greater levels of CatK protein than LNCaP cells, to a degree that CatK was detectable in the CM of PCa cells. PC3 cells are prostate cancer cells that are derived from bone metastatic site, also show higher expression levels of CatK compared to PrEC and LNCaP cells (Fig. 1c−f). These results indicate that the CatK expression correlates with PCa progression.

### CatK inhibitor diminishes PCa tumor invasion in vitro

**Fig. 2:**
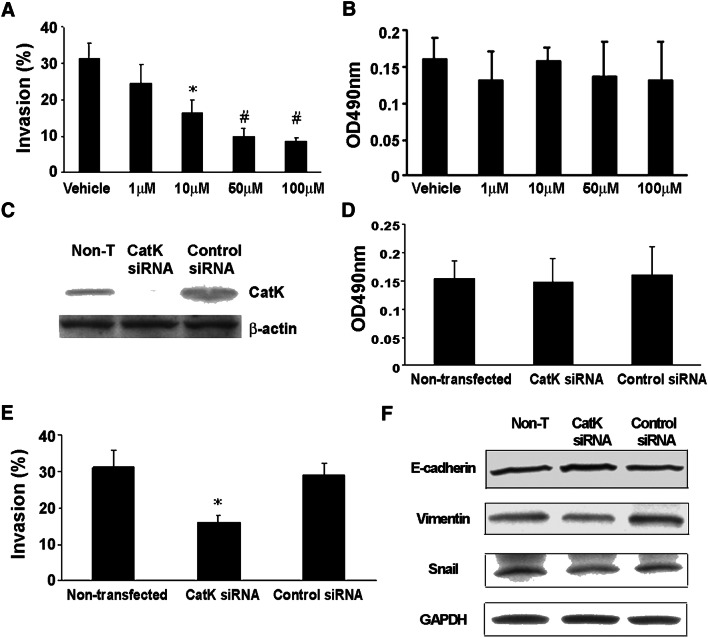
CatK inhibitor diminishes the invasiveness of C4-2B cells. **a** The in vitro invasion assay was performed using C4-2B cells cultured in 24-well transwell chambers (BD BioCoat Matrigel Invasion Chamber, BD Biosciences, MA), as directed by the manufacturer. Invasive ability was defined as the proportion of cells that penetrated the matrix-coated membrane divided by the number of cells that migrated through the uncoated membrane (baseline migration). The results are reported as the mean of triplicate assays. *P < 0.05 compared to the vehicle; ^#^*P *< 0.001 compared to the vehicle. **b** The C4-2B cells viability were examined by MTS assay. C4-2B cells were treated with various doses of CatK inhibitor (the doses that were used in the tumor invasion assay). We did not observe any significant differences among these cells in terms of cell viability. **c** The designed CatK siRNA or scrambled control siRNA were transfected into C4-2B cells using the transfection reagents. Cell lysates were collected for Western blot to confirm CatK expression was knocked down. **d** CatK expression knockdown cell viability was measured. There was no significant change among the parental cells and cells that transfected with CatK siRNA or control siRNA. **e** The ability of CatK expression knockdown cell invasion was examined. Knockdown CatK expression decreased C4-2B cell invasion. The results are reported as the mean of triplicate assays. *p < 0.01 compared to the control siRNA-transfected cells. **f** E-cadherin, Vimentin and Snail expression levels were detected by Western blot in CatK knockdown cells and the controls

To test the effects of CatK inhibitor on tumor growth and biological behavior in bone, the in vitro cell invasion and proliferation assays were performed. We observed that CatK inhibitor diminished invasiveness of C4-2B cells in vitro in a dose-dependent manner (Fig. 2a). Then, we examined the C4-2B cell viability (by MTS assay) that treated with various doses of CatK inhibitor (the same doses as used in the tumor invasion assay). We did not observe any significant differences among these cells in terms of cell viability (Fig. 2b), concluding that the CatK inhibitor has no significant toxicity for tumor cell growth. To further confirm the effects of CatK inhibitor in tumor growth, we used the C4-2B cells by knockdown CatK expression and control cells with regard to the ability of cell invasion and cell proliferation. We obtained similar results for CatK knockdown as using the CatK inhibitor (Fig. 2c–e). Moreover, to investigate possible mechanism of knockdown CatK expression diminish C4-2B cell invasion, we detected EMT hallmarks and found that E-cadherin was increased in CatK knockdown cells, and slight decrease of Vimentin and Snail, compared to the control or non-treat cells. This result suggests that altering the tumor cell invasiveness by CatK inhibitor could provide a novel therapeutic approach targeting the function of CatK in PCa skeletal metastases.

### CatK inhibitor diminishes PCa-CM-induced bone resorption in vitro

**Fig. 3:**
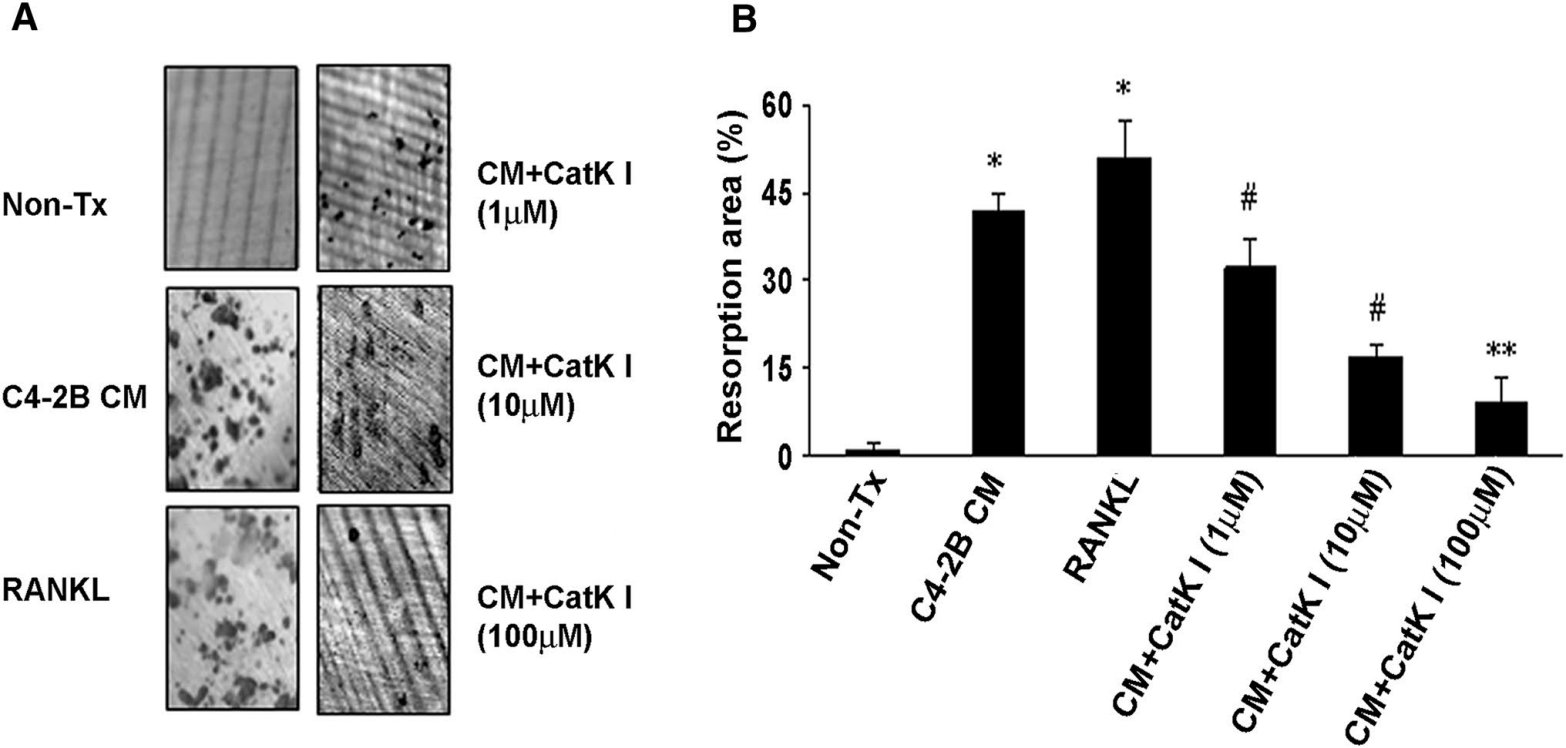
CatK inhibitor diminishes PCa-CM-induced bone resorption in vitro. **a** Representative images of resorption pits on dentin slices. **b** Samples were evaluated in triplicates. Results are reported as mean ± SD. *P < 0.001 compared to non-treatment (non-TX) group; ^#^*P *< 0.01 compared to C4-2B CM-treated group; **P < 0.001 compared to C4-2B CM-treated group. Data are a representative of 3 separate experiments

To evaluate the ability of the CatK inhibitor to diminish the PCa-CM-induced bone resorption, we first collected CM from C4-2B cell culture as previously described (Zhang et al. 2001). We seeded non-adherent primary murine bone marrow cells into 96-well plate which containing dentin slice. Soluble RANKL (50 ng/ml) or the concentrations of CatK inhibitor as indicated was added. We observed that C4-2B CM-induced bone resorption in this system. Coherent this induction was diminished by CatK inhibitor in a dose-dependent manner (Fig. 3).

### CatK inhibitor prevents the establishment and retards the progression of PCa tumor in murine bone

**Fig. 4:**
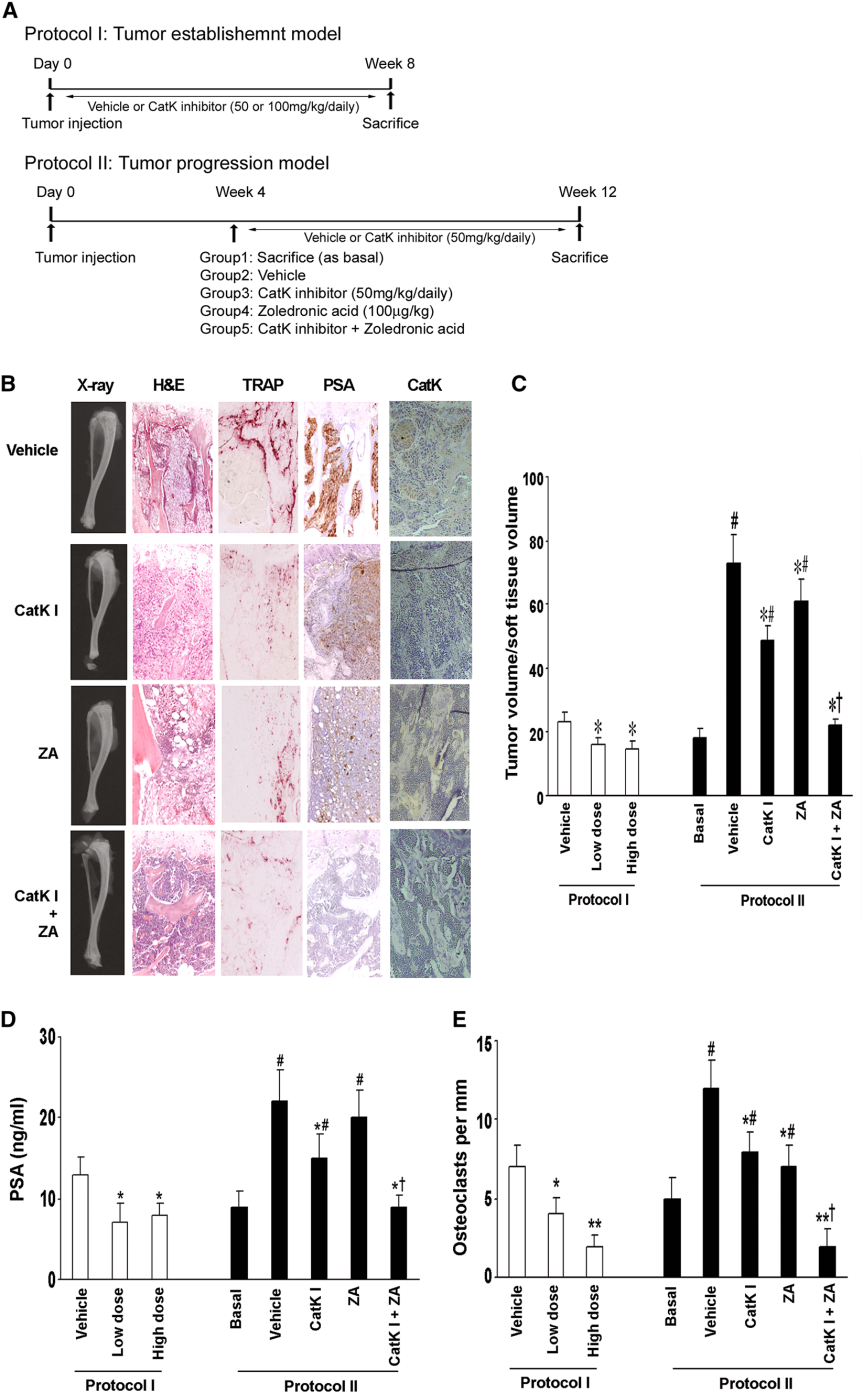
CatK inhibitor prevents the establishment and retards the progression of PCa tumor in mouse bone. **a** Schematic of experimental procedures to determine the effects of CatK inhibitor on the establishment and progression of prostate cancer. Mice were killed at the end of experiments. X-ray, H&E, and PSA were determined. **b** In this representative figure, note the area of osteolysis and osteosclerosis of the vehicle-treated mouse compared to the radiograph of the CatK inhibitor-treated mouse. PSA is strongly positive in all vehicle-treated mice compared to CatK inhibitor-treated mice. After CatK inhibitor treatment, TRAP-positive cells were apparently reduced in the tibiae compared to the vehicle group (for the tumor progression model, we observed the similar results). **c** Tumor volume versus non-bone soft tissue volume was measured by bone histomorphometry. **d** Serum PSA levels in the mice model were measured by ELISA and found CatK inhibitor decreased serum levels. **e** Osteoclast numbers per millimeter bone surface were quantified by bone histomorphometry. Results are reported as mean ± SD. *P < 0.01 compared to vehicle group; ^#^*P* < 0.01 compared to the basal group

**Fig. 5:**
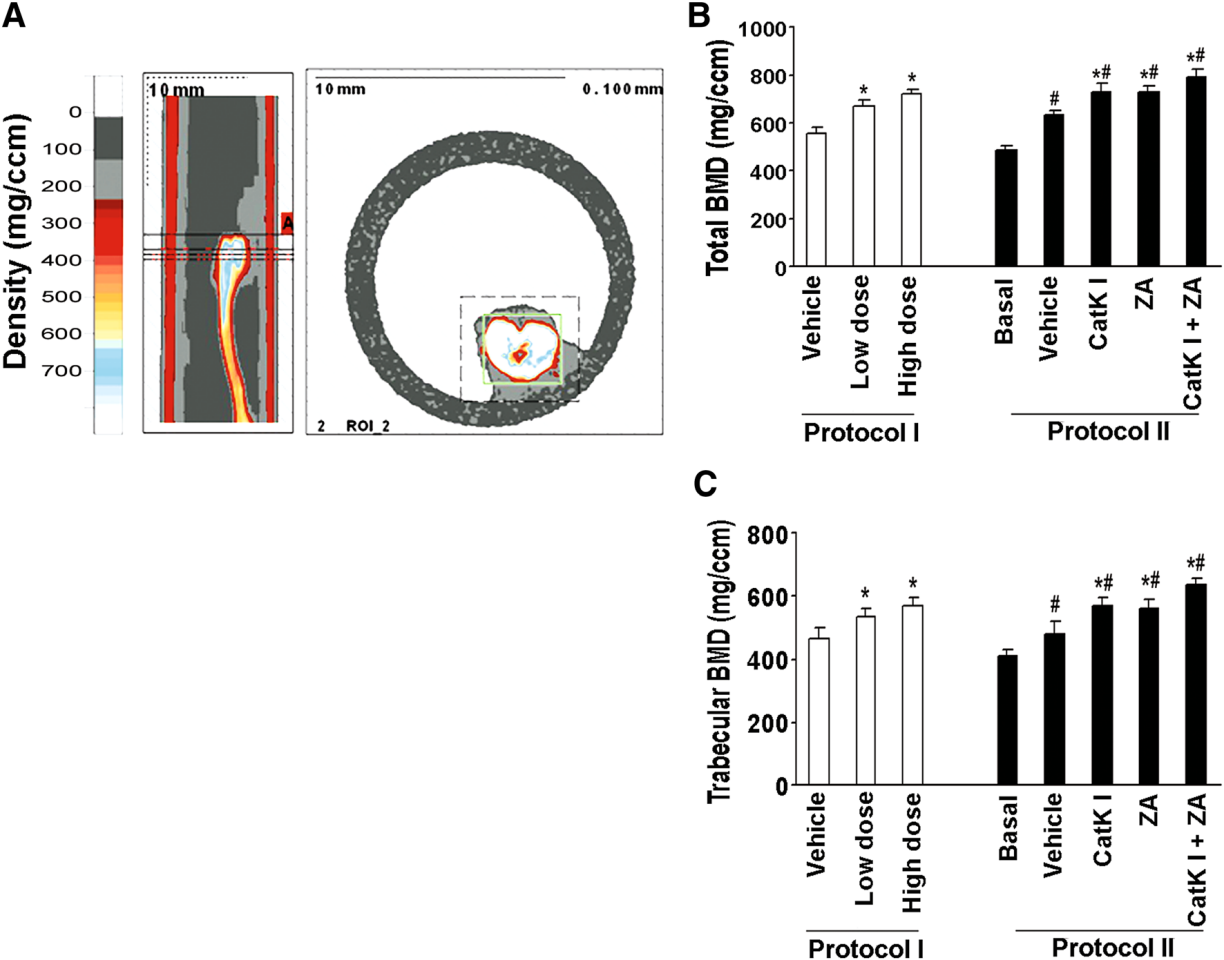
CatK inhibitor protects bone mineral density in mouse model. **a** Slices that were scanned by pQCT. The small box in the right panel indicates the tibia. The reference slice and the slices examined are indicated on a representative tibia. **b** Total bone mineral density detected by pQCT of tibiae from each group. **c** Trabecular bone mineral density detected by pQCT of tibiae from each group. *P < 0.01 compared to vehicle group; ^#^*P* < 0.01 compared to the basal group

**Fig. 6:**
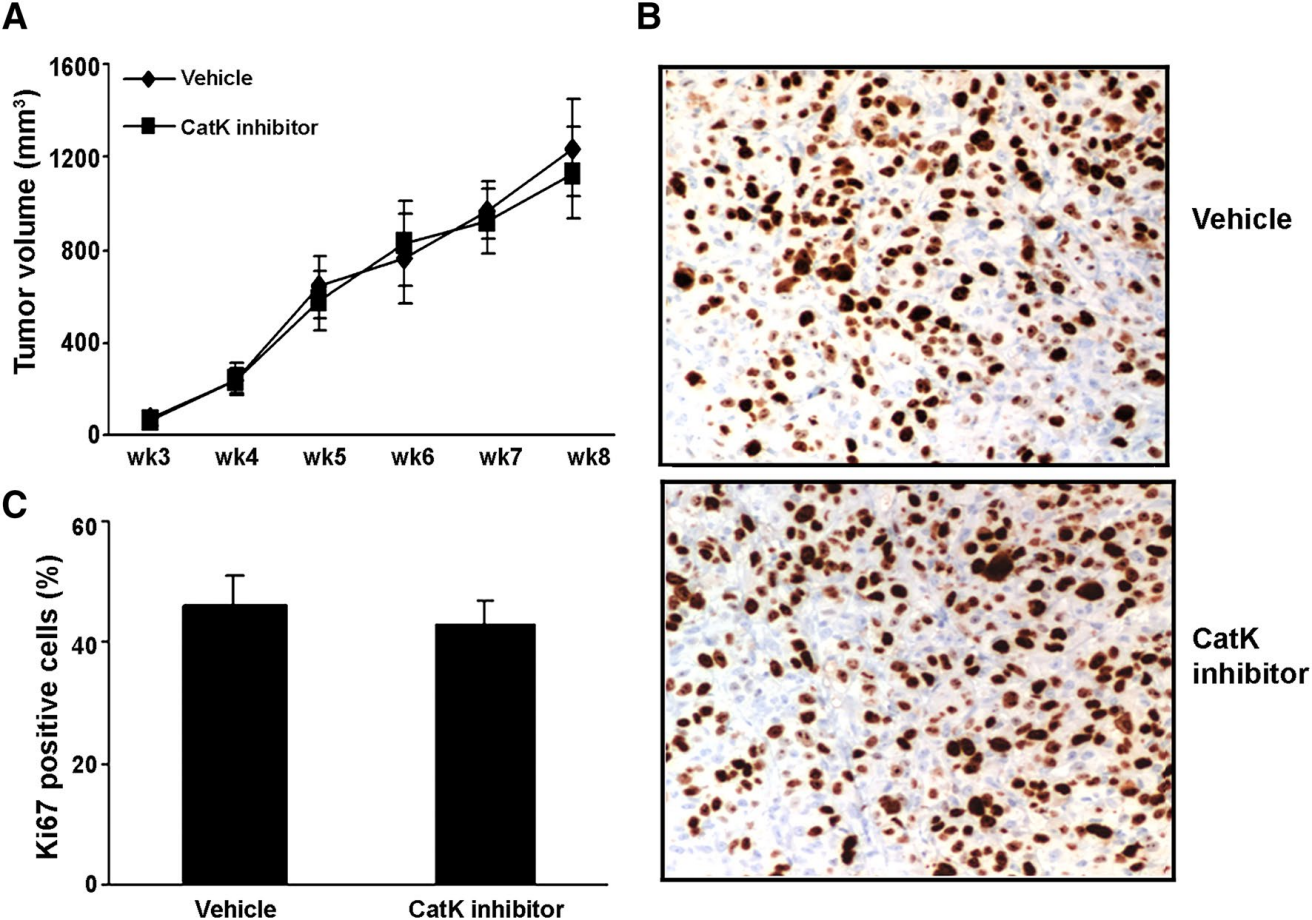
CatK inhibitor has no effect on subcutaneous tumor growth in vivo. **a** Tumor volume was monitored twice weekly by caliper measurements. **b** Subcutaneous tumor sections were immunohistochemically stained with Ki67 antibody. **c** Quantified data were determined by the number of Ki67-positive cells dividing the total numbers into five randomly selected fields under light microscopy (× 400)

To determine whether the inhibition of osteoclastogenic activity could prevent the establishment or retard the progression of PCa in mouse bone, we directly injected C4-2B cells into the tibia of SCID mice (*n* = 10/group). The experimental protocols are summarized in Fig. 4a. Furthermore, we injected C4-2B cells into subcutaneous sites of the same mice in protocol I to evaluate the differences in response in the bone versus a non-osseous site. The mice were administered orally either CatK inhibitor (50 mg/kg/day, or 100 mg/kg/day) or vehicle either at the time of tumor cell injection (Protocol I: tumor establishment model) or 4 weeks after tumor injection (Protocol II: tumor progression model). Mice were killed at the end of time points as indicated. We found that CatK inhibitor administration significantly inhibited the establishment and development of tumor in bone (Fig. 4b–e) (C4-2B caused dominantly osteolytic areas with some areas of osteoblastic activity). It significantly reduced osteoclast formation, serum PSA levels and CatK levels compared to the vehicle-treated animals. The inhibitory effects of the CatK inhibitor were enhanced in combination with ZA. These results indicated tumor burden was reduced. CatK inhibitor treatment also diminished the tumor-induced loss of bone mineral density based on the pQCT analysis (Fig. 5). Interestingly, we found CatK inhibitor had no significant effect on tumor growth at the subcutaneous sites (Fig. 6). Taken together, these results demonstrate that the effect of CatK inhibitor is specific to the bone microenvironment.

## Discussion

PCa skeletal metastases cause significant complications including severe bone pain, impaired mobility, pathological fracture, spinal cord compression and hypercalcemia (Park et al. 2018). Emerging evidence shows that cysteine proteases have been implicated in the progression of tumors and in various critical tumor biological processes, including tumor cell aberrant proliferation and apoptosis, tumor cell-induced angiogenesis, as well as invasion of surrounding tissues and metastasis by malignant cells, suggesting that they are relevant to drug targets for treating cancer (Turk et al. 2004). Cysteine cathepsins upregulation has been reported in many human tumors, including breast, lung, brain, gastrointestinal, PCa, and melanoma (Jedeszko and Sloane 2004). Recently, periostin (Postn) was identified as a direct molecular target for degradation by CatK. CatK knockout mice show that CatK deletion increases Postn and β-catenin expression in vivo, particularly at the periosteum. In turn, Postn deletion selectively abolishes cortical, but not trabecular, bone formation in CatK-deficient mice. These findings point to CatK not only playing a major role in bone remodeling but also modulating modeling-based cortical bone formation (Bonnet et al. 2017). Using a prostate carcinoma cell line engineered to overexpress CatK, the light-activated inhibitor effect of CatK on CatK-mediated collagen I degradation can be quantified and even instantly monitored by assessing the living tumor spheroids formation (Herroon et al. 2016).

Several lines of evidence have shown that osteoclastic lesions are important for the development of bone metastases (Fazzalari et al. 2001). Anti-resorptive approaches such as administration of bisphosphonates or anti-PTHrP neutralizing antibody have been reported in breast cancer models to be able to block the tumor expansion in bone (Guise et al. 1996; Sasaki et al. 1995). Bisphosphonates could prevent bone loss by inducing cell death of osteoclasts. However, bisphosphonates have several disadvantages such as numerous side effects (upper gastrointestinal irritation, fever, pain, delayed fracture healing, etc.) and a very long half-life in the human body (> 10 years), implying a serious concern (Bromme et al. 2016; Grabowska et al. 2005). Furthermore, it has been recently reported that bisphosphonates are capable of causing osteonecrosis of the jaws (Marx 2003; Melo and Obeid 2005; Ruggiero et al. 2004), inducing inflammation and rupturing atherosclerotic plaques in apolipoprotein-E null mice (Shimshi et al. 2005; Zhao et al. 2018). Therefore, new treatments are urgently needed for patients with bone metastases and those at high risk of developing bone metastases (Bromme et al. 2016; Munari et al. 2017).

In current experiments, we have found that PCa cell lines, C4-2B, LNCaP, and PC3 expressed CatK mRNA and protein, yet poorly in the prostate epithelial cells (PrEC). CatK expression was greater in metastatic PCa cells C4-2B and PC3 cells compared to nonmetastatic LNCaP cells both at mRNA level and protein level. We also found that CatK was expressed at a low level in the normal prostate, increased in the primary prostate tumor, and at a high level in a skeletal metastatic tumor by immunohistochemistry of tissue samples (tissue microarray). Altogether, these results pinpoint the possibility that the selective CatK inhibitor targets not only bone cells but also tumor cells.

The CatK inhibitor used in this study is an enzyme inhibitor that should not adversely affect the tumor cell, simultaneously keep the interplay between bone resorbing and bone forming cells intact. Accordingly, we examined the viability of C4-2B cells that were treated with CatK inhibitor at doses as indicated. We did not observe any significant differences. Interestingly, CatK inhibitor diminished invasion of C4-2B cells in vitro. Then CatK expression was blocked in PCa cells using CatK siRNA and further confirmed the results of using a CatK inhibitor in regard to cell invasion. Ideally, it should inhibit collagen break-down during bone resorption, but leave the bone formation process intact, which could lead to an increase in bone substance. In this study, we found that the CatK inhibitor dose-dependently diminished PCa-CM-induced bone resorption in vitro. These results indicate that CatK may play an important role during PCa metastasis and progression.

To further determine the effects of CatK inhibitor in vivo, we observed the CatK inhibitor could prevent the establishment of PCa tumor in mouse bone using tumor establishment model, and retard tumor progression in murine bone using tumor progression model. As expected, the CatK inhibitor increased bone mineral density in both mouse models. We also found the CatK inhibitor had no effect on subcutaneous tumor growth. These results provide strongly support that CatK is a key factor specific in PCa skeletal metastasis and PCa-induced bone lesions in vivo.

## Conclusion

Our data showed for the first time that the selective CatK inhibitor reduced PCa-induced bone lesions, diminishes tumor burden in bone and increases bone formation. The inhibitory effects of the CatK inhibitor were enhanced in combination with ZA. These results make the CatK inhibitor a potential agent treating bone diseases including PCa patients at advanced disease stage. This novel and unique targeting strategy has established the rationale to inhibit tumor-induced bone resorption at bone metastatic sites.

## Abbreviations

CatK: Cathepsin K
OPG: Osteoprotegerin
PCa: Prostate cancer
PTHrP: Parathyroid hormone-related protein
ZA: Zoledronic acid
RANKL: Receptor activator of nuclear factor kappaB ligand
Non-Tx: Non-treatment
